# QT Prolongation and Torsade De Pointes After Catheter Ablation for Persistent Atrial Fibrillation in a Patient With Tachycardia-Induced Cardiomyopathy: A Case Report

**DOI:** 10.7759/cureus.61640

**Published:** 2024-06-04

**Authors:** Daiki Yamashita, Naoki Fujimoto, Yoshihiko Kagawa, Satoshi Fujita, Kaoru Dohi

**Affiliations:** 1 Department of Cardiology and Nephrology, Mie University Graduate School of Medicine, Tsu, JPN

**Keywords:** atrial fibrillation, catheter ablation, qt prolongation, torsade de pointes, tachycardia-induced cardiomyopathy

## Abstract

Atrial fibrillation (AF) is the most common cause of tachycardia-induced cardiomyopathy (TIC). A 75-year-old woman was referred to our hospital for catheter ablation for persistent AF. On admission, transthoracic echocardiography (TTE) revealed diffuse left ventricular (LV) hypokinesis, which was suspected to be due to TIC. Catheter ablation was performed on the fifth day of hospitalization, and Torsade de Pointes (TdP) appeared on the sixth day. The serum concentration of bepridil and potassium was below the reference level. An electrocardiogram revealed marked QT prolongation, giant-negative T waves, and T-wave alternans on the seventh day of hospitalization. Cardiac magnetic resonance imaging with no contrast indicated diffuse mild LV hypokinesis, mild prolonged native T1, and no evidence of myocardial edema at T2. Coronary angiography revealed normal coronary arteries, and the ergonovine stress test results were negative. The results for five long QT syndrome susceptibility genes, including the three major genes, were negative. Subsequently, QT prolongation, giant-negative T waves, and LV dysfunction improved without treatment. This case report highlights the importance of risk management for AF patients with TIC scheduled for catheter ablation and carefully evaluating the risks of QT prolongation. Moreover, patients with TIC can experience marked QT prolongation and TdP during the perioperative period of catheter ablation. Therefore, caution should be required.

## Introduction

Cardiomyopathies, such as tachycardia-induced cardiomyopathy (TIC), are a heterogeneous group of myocardial diseases and an important cause of heart failure (HF) [1]. TIC is a condition characterized by decreased left ventricular (LV) contractility due to prolonged tachycardia, such as rapid and/or irregular ventricular rate [2]. Atrial fibrillation (AF) is the most common cause of TIC [3]. The resolution of tachycardia can result in an improvement in LV contractility. TIC is a significant cause of HF because it is possibly reversible after suitable heart rate or rhythm control. In a previous large TIC cohort, treatment of the arrhythmia with rhythm control, such as cardioversion or radiofrequency catheter ablation, led to improved echocardiographic LV function [4]. Even after arrhythmias are treated and LV contractility improves, the risks of HF hospitalization and sudden cardiac death due to the recurrence of arrhythmias remain [5,6]. In addition, data on the long-term outcomes of TIC patients are limited [6,7]. TIC remains an unexplored disease, its clinical course is not uniform, and specific diagnostic criteria have not been established. Here, we report a case of QT prolongation and Torsade de Pointes (TdP) after catheter ablation for persistent AF in a patient with TIC.

## Case presentation

**Figure 1:**
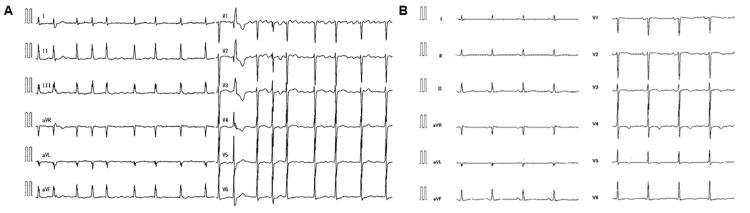
Electrocardiogram ECG showing AF rhythm, PVC, no ST-T segment changes, and mild QT prolongation (QTc: 450 ms; the average of three beats), with a heart rate of 104 bpm at the first examination (A). After catheter ablation, the ECG showed sinus rhythm, mild QTc prolongation (470 ms), a mild negative T wave in leads V3, V4, and V5, and a heart rate of 68 bpm (B). ECG, electrocardiogram; AF, atrial fibrillation; PVC, premature ventricular contraction; bpm: beats per minute.

A 75-year-old woman with a medical history of asthma presented to a local clinic with AF documented during a medical checkup. The patient had previously undergone percutaneous coronary intervention and had no family history of heart disease or sudden death. Her medications included edoxaban 60 mg per day, aspirin 100 mg per day, and valsartan 80 mg per day. A Holter electrocardiogram (ECG) was performed in 24 hours, which showed an AF burden of 100% and a premature ventricular contraction (PVC) burden of 13%. Bepridil 100 mg per day was then initiated. She was referred to our hospital with palpitations and chest discomfort. The ECG indicated AF rhythm, PVC, no ST-T segment change, and mild QT prolongation (QTc, 450 ms; the average of three beats), with a heart rate of 104 beats per minute (bpm) (Figure 1A). Transthoracic echocardiography (TTE) indicated normal LV function with an LV ejection fraction (LVEF) of 60%. Catheter ablation was scheduled after obtaining informed consent from the patient.

One month after the initial visit, the patient presented to our hospital two days before her scheduled admission owing to worsening symptoms. The physical examination revealed a body temperature of 36.3°C, a heart rate of 120 bpm, blood pressure of 124/94 mmHg, and an oxygen saturation level of 95%. The chest radiography revealed mild congestion with a cardiothoracic ratio of 62%. There were no remarkable changes in the ECG findings, except for a higher heart rate of 120 bpm and frequent PVCs compared with the initial visit. The laboratory test results revealed slightly elevated troponin-I levels of 23.4 pg/mL (normal range: below 15.6 pg/mL) and B-type natriuretic peptide (BNP) levels of 238.3 pg/mL, as well as decreased potassium levels of 2.7 mmol/L. The BNP values remained unchanged compared to those at her initial visit. TTE indicated diffuse LV hypokinesis with an LVEF of 40%, which was suspected to be due to tachycardia (Table 1). The patient was admitted to our hospital on the same day, and potassium correction was initiated.

**Table 1:** The patient’s laboratory findings

	Values	Units
White blood cell	8250	/μL
Hemoglobin	14.4	g/dL
Platelet	27.2×10^4^	/μL
Blood urea nitrogen	13.4	mg/dL
Creatinine	0.62	mg/dL
Sodium	144	mmol/L
Potassium	2.7	mmol/L
Magnesium	2.0	mg/dL
C-reactive protein	1.06	mg/dL
B-type natriuretic peptide	238.3	pg/mL
Troponin-I	23.4	ng/mL
Creatinine kinase	66	IU/L

**Figure 2:**
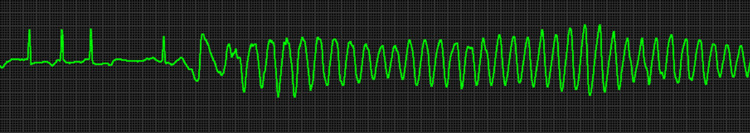
Torsade de Pointes recorded by monitor electrocardiogram On the sixth day of hospitalization, Torsade de Pointes levels were measured.

On the fifth day of hospitalization, catheter ablation was performed with pulmonary vein isolation, left atrial posterior isolation, and superior vena cava (SVC) isolation, owing to the presence of trigger premature atrial contractions in SVC. Following ablation, the ECG revealed sinus rhythm, a mild QTc prolongation (470 ms), a mild negative T wave in leads V3, V4, and V5, and a heart rate of 68 bpm (Figure 1B). On the sixth day of hospitalization, TdP appeared and soon resolved spontaneously (Figure 2), and the following day, TdPs appeared several times. Although the patient took bepridil 100 mg per day one month before hospitalization, the serum concentration was below the reference level (146 ng/mL, normal range: 250-800 ng/mL). Her serum potassium concentration was 4.0 mmol/L.

**Figure 3:**
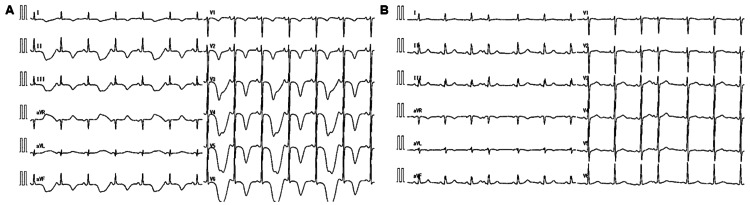
Electrocardiogram On the seventh day of hospitalization, ECG indicated marked QT prolongation (QTc; 750 ms), giant-negative T waves, and T-wave alternans (A). An ECG performed a month and a half after discharge from the hospital revealed improvement in QT prolongation (QTc; 400 ms) and the disappearance of the negative T wave (B). ECG, electrocardiogram.

Bepridil was discontinued, magnesium sulfate and isoproterenol were administered, and a temporary pacemaker was inserted to maintain her heart rate at 80 bpm. On the seventh day of hospitalization, the ECG indicated marked QT prolongation (QTc: 750 ms), giant-negative T waves, and T-wave alternans (Figure 3A).

**Figure 4:**
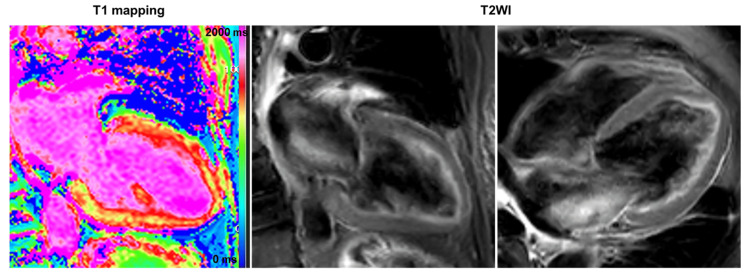
Cardiac magnetic resonance imaging (CMRI) on day 12 The CMRI indicated diffuse LV mild hypokinesis and prolonged native T1 (1365 ms; reference level 1314 ± 29 ms). Moreover, T2 in CMRI showed no obvious high-signal areas within the LV myocardium, and global T2 was 51 ms (reference level 46 ± 4 ms) with no obvious prolongation. There was no evidence of myocardial edema at T2.

The TTE revealed no remarkable changes compared to admission. On the 12th day of hospitalization, cardiac magnetic resonance imaging (CMRI) with no contrast indicated mild prolonged native T1 (1365 ms; normal range: 1314 ± 29 ms) and no evidence of myocardial edema at T2 (Global T2; 51 ms) (Figure 4). On the 20th day of hospitalization, coronary angiography revealed normal coronary arteries, and the ergonovine stress test results were negative.

Fortunately, the QT prolongation and giant-negative T waves improved, and the patient was discharged on day 21. ECG and TTE performed six weeks after discharge from the hospital revealed normalization of QTc to 400 ms, disappearance of the negative T wave (Figure 3B), and improvement in LV wall motion. A Holter ECG was also repeated in 24 hours. The result showed no recurrence of AF or other supraventricular arrhythmias. PVC burden decreased to 0.01%. Considering the possibility of concealed long QT syndrome (LQTS), genetic testing was performed. The results for five LQTS susceptibility genes, including the three major genes (*KCNQ1* (LQT1), *KCNH2* (LQT2), and *SCN5A* (LQT3)), were negative.

## Discussion

In the present patient, LV wall motion was impaired over time, probably owing to sustained tachycardia, which improved after rhythm control by catheter ablation. These findings are consistent with the clinical course often observed in patients with TIC. Changes in K-channel expression in the ventricular myocardium cause QT prolongation in rabbits with pacing-induced HF and TIC [8]. In our patient, possible changes in K-channel expression in the LV myocardium may have resulted in QT prolongation.

Bepridil is a class IV oral antiarrhythmic drug (AAD) that is mainly effective in blocking calcium and potassium currents [9]. QT prolongation and TdP were known side effects of bepridil. Bepridil is recommended as the standard AAD for rhythm control in persistent AF in the Japanese pharmacotherapy guidelines for AF [10]. We initially suspected LQTS secondary to bepridil or hypokalemia; however, blood bepridil levels were below the reference values, and serum potassium levels were within normal limits. Blood bepridil concentration appeared to positively correlate with QT prolongation, and the lower limit of blood bepridil in patients with TdP is reportedly 500 ng/mL [11]. These findings suggest that bepridil was not associated with LQT in the present patient.

Notably, takotsubo cardiomyopathy may have been a differential diagnosis in this patient. We observed marked QT prolongation and giant-negative T waves on the ECG, with no coronary artery disease. In typical cases of takotsubo cardiomyopathy, hypercontraction at the cardiac base and reduced wall motion at the apex are observed on TTE. Moreover, a high T2 value that reflects myocardial edema is observed, particularly in the LV segment with depressed wall motion on CMRI [12].

It is important to recognize that one of the important triggers for TdP in AF is the underlying coronary artery disease (CAD). The relationship between AF and CAD is well established but often overlooked, as they share similar risk factors and common pathophysiological characteristics [13-15]. The two form a vicious cycle wherein one disease promotes the other. We initially speculated that the cause of LV dysfunction was TIC. Therefore, we did not perform any examinations of the coronary arteries in this case. Following the onset of TdP, we performed coronary angiography and the ergonovine stress test, both of which yielded negative results. Therefore, it is crucial to take into account the potential complications of both TIC and CAD in patients with AF who also have LV dysfunction.

## Conclusions

There are limited reports on TdP during the perioperative period of catheter ablation in patients with TIC. This case report highlights the importance of risk management for AF patients with TIC scheduled for catheter ablation. During the perioperative period of catheter ablation, careful evaluation of the risks of QT prolongation is required, as there are multiple triggers for QT prolongation, such as TIC, HF, catheter ablation, sinus conversion, antiarrhythmic drugs, electrolyte abnormalities, vasospastic angina, and other cardiomyopathies. TIC can potentially cause life-threatening arrhythmias, even though it is commonly considered to have favorable outcomes in the absence of recurrent arrhythmias. Patients with TIC can experience marked QT prolongation and TdP during the perioperative period of catheter ablation. Therefore, caution should be required.
